# A Polyoxoniobate/g-C_3_N_4_ Nanoporous Material with High Adsorption Capacity of Methylene Blue from Aqueous Solution

**DOI:** 10.3389/fchem.2018.00007

**Published:** 2018-01-31

**Authors:** Qiuyan Gan, Weilong Shi, Yanjun Xing, Yu Hou

**Affiliations:** ^1^College of Chemistry, Chemical Engineering and Biotechnology, Donghua University, Shanghai, China; ^2^Institute of Functional Nano and Soft Materials, Soochow University, Suzhou, China

**Keywords:** polyoxoniobate, carbon nitride, methylene blue, adsorption, nanoporous material

## Abstract

A polyoxoniobate/g-C_3_N_4_ nanoporous material with functional groups has been synthesized by using carbon nitride (g-C_3_N_4_) and hexaniobate (K_8_Nb_6_O_19_·10H_2_O, abbreviated as NbO) as precursors. The structure and compositions of the as-prepared nanomaterials were characterized by XRD, FT-IR, FESEM, TEM, and XPS. These two kinds of materials interact with each other forming a hybrid composite, which can be used as an adsorbent for removing a cationic dye (methylene blue, MB) from wastewater with excellent adsorption capacity. Furthermore, parameters that can affect adsorption process including initial dye concentration, pH and temperature were investigated by bath adsorption experiments. The results indicated that the maximum adsorption capacity of NbO/g-C_3_N_4_ can reach up to 373.1 mg g^−1^. Moreover, the equilibrium experiment data fitted Langmuir isotherm well and the adsorption kinetics showed that the pseudo second order model can satisfyingly described MB adsorption kinetics. The thermodynamic analysis indicated that the adsorption was endothermic and spontaneous.

## Introduction

Organic dyes has been a vital environmental pollution source since various industries use it in many fields, such as textile, leather, printing, dyestuff, and cosmetics (Liu X. X. et al., 2016; Cai et al., 2017). Among all dyes, methylene blue (MB), is one of the most common dying pollutants (Xu et al., 2017). The excessive emission of MB in the environment will induce eye burns, mental confusion, shock, and vomiting (Xu et al., 2017). Hence, it is exigent to develop effective materials to remove the MB from waste water.

In order to eliminate organic dyes from waste water, several methods including adsorption (Haque et al., 2011; Wang et al., 2016; Yan et al., 2016), photo-degradation (Kumar et al., 2014; He et al., 2015; Xin et al., 2017), chemical oxidation (Osugi et al., 2009), and biological treatment (Zhang et al., 2014) have been used. Compared to others, adsorption is considered as a most effective method due to its simple design and operation, high efficiency, and economy (Haque et al., 2011). To promote the removal efficiency, a variety of adsorbents have been synthesized for the removal of MB, such as activated carbon (Bestani et al., 2008), graphene (Liu et al., 2012), magnesium oxide (Moussavi and Mahmoudi, 2009; Hu et al., 2010), and polymer (Xiao et al., 2015). Recently, graphitic carbon nitride (g-C_3_N_4_), a structurally graphite-like material, has been widely studied in photocatalytic hydrogen production (Wang et al., 2012) and removing water pollutants (Wang et al., 2013) due to its low cost, outstanding physicochemical properties and excellent chemical and thermal stability. However, reports on g-C_3_N_4_ as adsorbent to adsorb dyes was rare, mainly due to its low surface area, very limited functional groups and less binding sites. Peng and coworkers synthesized a series of mesoporous carbon nitride to adsorb MB and the adsorbents exhibited noteworthy effects (Peng et al., 2017). This study showed that g-C_3_N_4_ is able to remove organic dyes from the aqueous environment, and it is also a promising material for adsorption.

Polyoxometalates (POMs) has attracted extensive attention owing to their special structural properties and extensive applications in catalysis, medicine, and materials. They can also combine with other materials to modify the structure and properties of the materials (Troupis et al., 2002). Recently, a few studies on incorporating tungsten- and molybdem- containing POMs into g-C_3_N_4_ have been reported (Li et al., 2014; Long et al., 2014; He et al., 2015; Liu J. H. et al., 2016; Zhao et al., 2017), such as PW_12_O_40_/g-C_3_N_4_, PMo_12_O_40_/g-C_3_N_4_, Co_4_PW_9_O_34_/C_3_N_4_, g-C_3_N_4_/PW_11_O_39_, FePW_4_O_24_/g-C_3_N_4_, and PMo_10_V_2_O_40_/C_3_N_4_. Results indicated that the polyanionic, highly redox-active and acidic properties of POMs significantly modified the properties of g-C_3_N_4_ and these hybrid materials showed great photocatalytic performances on degradation of dyes, water oxidation, and hydrocarbon oxidation. Polyoxoniobates (PONbs), a branch of POMs, were different from W-, Mo-, and V- containing POMs in both synthesis and aqueous solution behaviors. They have unique properties including high specific surface area, high charge, high alkalinity, and photocatalytic activity. These properties endow them with applications for photocatalysis of water splitting, based-catalyzed decomposition of contaminants, and biological activity (Wu et al., 2015). Thus, it would be very interesting to combine polyoxoniobate with g-C_3_N_4_ to see if PONb has different influence on the photocatalytic performance. Surprisingly, the prepared material shows great adsorption capacity for MB in aqueous solution when we attempt to use it as photocatalyst for degradation of dyes. To the best of our knowledge, no such material have been synthesized and tested for the adsorption of organic dyes.

In this study, we used a facile hydrothermal treatment method to synthesize NbO/g-C_3_N_4_ material with porous structure by using K_8_Nb_6_O_19_·10H_2_O (NbO) and g-C_3_N_4_ as precursors. And we choose MB as an adsorbate to explore the adsorption capacity of NbO/g-C_3_N_4_ material. The structural and chemical property of NbO/g-C_3_N_4_ was investigated, and the results showed that both of them have important influence on the performance of adsorption dyes. In addition, the effects of different parameters on MB adsorption by NbO/g-C_3_N_4_ were also investigated. We also studied the thermodynamics, adsorption isotherms, and kinetics of adsorption process. The results revealed that NbO/g-C_3_N_4_ is an efficient adsorbent for removing MB from waste water.

## Experimental section

### Materials and methods

Anhydrous niobium oxide (Nb_2_O_5_, ≥99.5%), potassium hydroxide (KOH, ≥85%), methylene blue (MB) were purchased from Sinopharm Chemical Reagent. Melamine (99%) was obtained for Sigma-Aldrich. All the chemicals were used as received without further purification. The synthesis of NbO/g-C_3_N_4_ hybrid was completed by hydrothermal treatment method. Firstly, g-C_3_N_4_ was synthesized by direct heating melamine. Briefly, 5 g melamine power was placed into an alumina crucible with a cover and heated to 550°C for 2 h at a rate of 10°C/min in a muffle furnace under air. After cooling to room temperature, the resulting yellow sample was obtained. NbO was prepared according to the previous literature and its identity was further confirmed by IR spectrum (Liu et al., 2015). Two hundred milligrams of NbO was dissolved in 15 mL distilled water, and then 184 mg of as-prepared g-C_3_N_4_ were added into the aqueous solution. The suspension was ultrasound continuously for 10 h. Finally, the resulting mixture transfer into a Teflon-lined stainless autoclave at 180°C for 24 h. The product was centrifuged, washed with distilled water, and dried at 60°C for 12 h.

### Characterizations

The morphology of the samples was observed by using a field emission scanning electron microscope (FESEM, S-4800) and a transmission electron microscopy (TEM, JEM-2100) operating at 200 kVX-ray diffraction studies (XRD, D/max-2550VB+/PC) was carried out to determine the phase composition of the samples. The functional groups on the NbO/g-C_3_N_4_ surface were obtained on Fourier transform infrared spectroscopy (FTIR, Nicolet iS5). The FTIR spectra were recorded in the range 400–4000 cm^−1^ using KBr pellets. The real chemical composition of the products was determined using X-ray photoelectron spectroscopy (XPS, ESCA Lab250). The concentration of the dye was determined on UV-Vis spectrophotometer (UV 210).

### Adsorption experiments

Adsorption experiments were carried out in Erlenmeyer flask (250 mL), where solution of MB (200 mL) with initial dye concentration of 10–40 mg L^−1^ was placed. The flask with MB solution was sealed and placed in a constant temperature bath and agitated by magnetic stirrer. In order to investigate the effect of temperature, the experiments were carried out at five different temperatures, that is, 20, 30, 40, 50, and 60°C. When the desired temperature was reached, about 0.01 g of NbO/g-C_3_N_4_ was added into flask. The pH effect was also studied. Before mixing with the adsorbent, various pH levels (3.0–11.5) of the dye solution were adjusted by adding a few drops of diluted hydrochloric acid (0.1 M HCl) or sodium hydroxide (0.1 M NaOH).

At the end of the equilibrium period, aqueous sample (3 mL) was taken from the solution and then filtered by syringe filter. The concentration of MB in solution was determined by UV-Vis spectrophotometer at a wavelength of 664 nm. The amount of MB adsorbed at equilibrium *q*_e_ (mg g^−1^) was calculated by following equation:

(1) $$\begin{array}{r} q_{\text{e}}=\left(C_{0}-C_{\text{e}}\right)V/m \end{array}$$

Where *C*_0_ (mg L^−1^) is the initial MB concentration, *C*_e_ (mg L^−1^) is the MB concentration at equilibrium, *V* (L) is the volume of MB solution, and *m* (g) is the mass of the adsorbent.

For the kinetic experiments, 0.01 g of NbO/g-C_3_N_4_ was added into 200 mL of MB solution at different initial concentrations and temperatures. The adsorption capacity *q*_t_ (mg g^−1^) at different adsorb times was calculated by the following equation:

(2) $$\begin{array}{r} q_{\text{t}}=\left(C_{0}-C_{\text{t}}\right)V/m \end{array}$$

where *C*_t_ (mg L^−1^) represented the concentration of MB at any time *t*.

## Results and discussion

### Characterizations of the adsorbent

**Figure 1:**
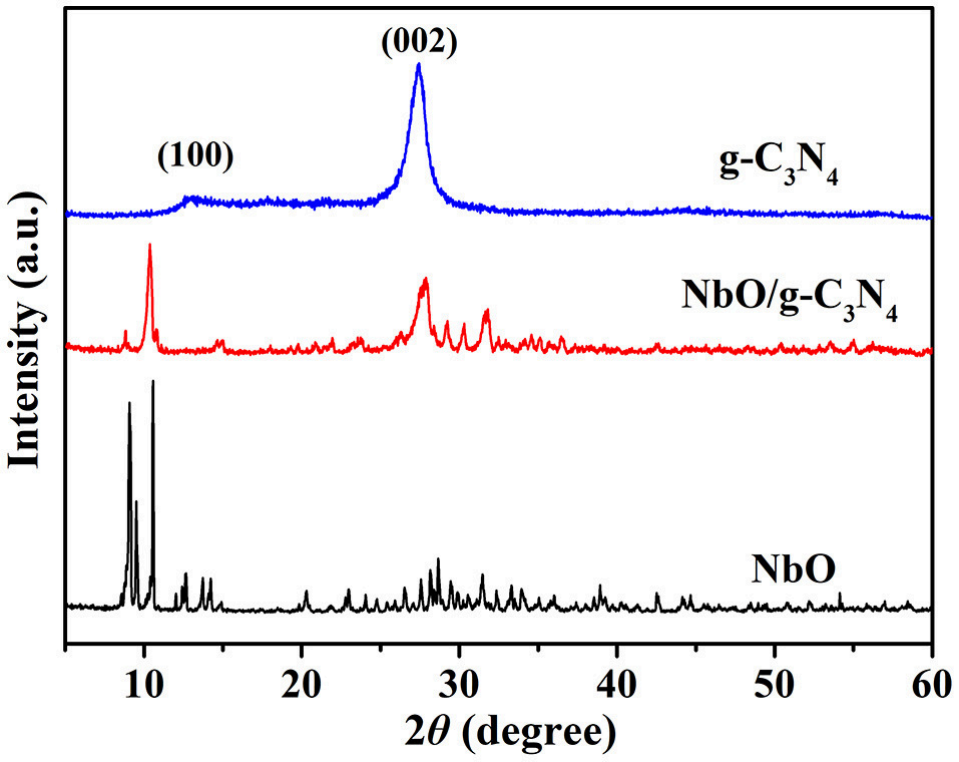
XRD powder patterns of g-C_3_N_4_, NbO/g-C_3_N_4_, and NbO.

XRD powder patterns were carried out to analyze the structures of the prepared g-C_3_N_4_, NbO, and NbO/g-C_3_N_4_ materials (see Figure 1). Two characteristic peaks at 13.1° and 27.4° were indexed to (100) and (002) planes of pristine g-C_3_N_4_ (Zhang et al., 2016), and these two peaks can be ascribed to the in-plane structural packing motif of tris-triazine units and the interlayer stacking of aromatic systems, respectively (Zhang et al., 2009). The bottom XRD pattern of NbO could be assigned to the phase of pure NbO (JCPDS 14-0288). The spectrum of NbO/g-C_3_N_4_ composite shows the diffraction peaks of both g-C_3_N_4_ and NbO. This indicates that NbO has successfully reacted with g-C_3_N_4_ sheets and both of them are good in the hybrid composite.

**Figure 2:**
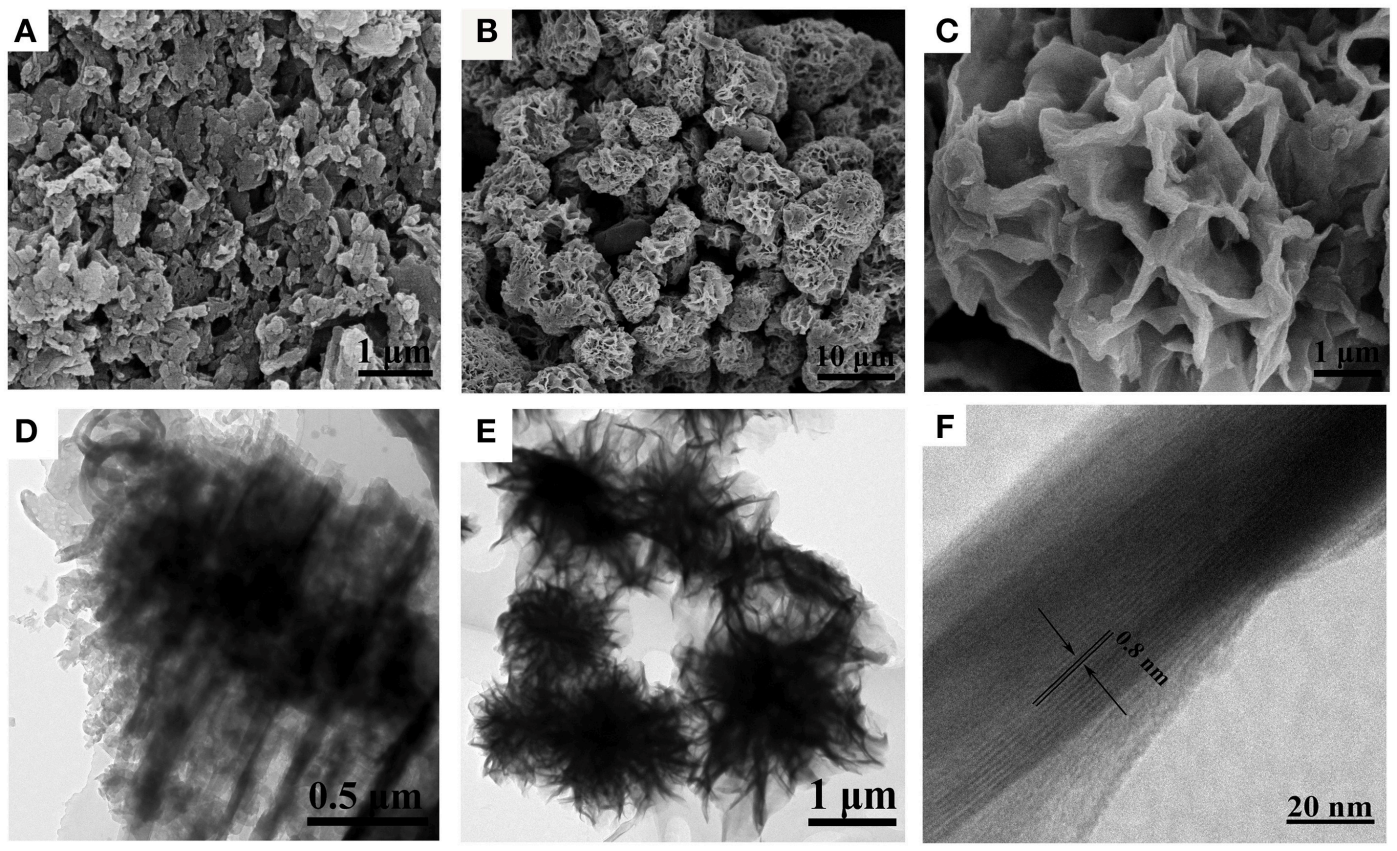
FESEM images of g-C_3_N_4_ **(A)**, NbO/g-C_3_N_4_ **(B,C)**, and TEM images of g-C_3_N_4_ **(D)**, NbO/g-C_3_N_4_ **(E,F)**.

The morphology and structures of the samples were characterized by using both FESEM and TEM (Figure 2). Figure 2A shows typical FESEM image of sheet structure of pristine g-C_3_N_4_. It can be seen that the morphology of g-C_3_N_4_ was irregular and the sizes were different with each other. The FESEM image (Figure 2B) of NbO/g-C_3_N_4_ composite was like a heap of flower-like spheres, which were assembled from plenty curved nanosheets with varied sizes. Moreover, the FESEM image (Figure 2C) shows clearly surface structure of the composite. Closer observation demonstrated that the microspheres have highly uniform structure and highly porous texture. The structure of NbO/g-C_3_N_4_ was completely different with g-C_3_N_4_ since the hydrothermal treatment with NbO at 180°C could significantly modified the structure of g-C_3_N_4._ The changes of the structure can be further confirmed by the TEM images. Figure 2D shows the pristine bulk g-C_3_N_4_ with obvious layer structure. In contrast, TEM image of NbO/g-C_3_N_4_ (Figure 2E) reveal the nanosheets are crumpled, and Figure 2F exhibits that the average thickness were ~0.8 nm in the nanosheets, which further indicated its ultrathin thickness.

**Figure 3:**
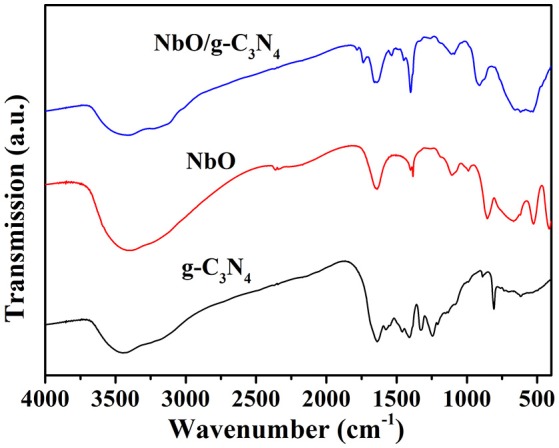
FTIR spectra of g-C_3_N_4_, NbO, and NbO/g-C_3_N_4_.

The FTIR spectra of prepared g-C_3_N_4_, NbO, and NbO/g-C_3_N_4_ composite are shown in Figure 3. The Nb-O band in the composite is clearly visible in the region 515–900 cm^−1^, although there is a broad band in the range from 515 to 663 cm^−1^ compared to the peaks at 507 and 643 cm^−1^ of NbO. In the spectrum of g-C_3_N_4_, the broad band around 3150 cm^−1^ is indicating N-H stretching vibrations, the peaks at 1626 and 1235 cm^−1^ were corresponding to the C = N and C-N stretching vibrations, respectively. The peak at 805 cm^−1^ was related to the s-triazine ring vibrations (Kumar et al., 2014). Therefore, all characteristic peaks of NbO and g-C_3_N_4_ were observed in NbO/g-C_3_N_4_ composite sample. The UV-vis absorption spectra of g-C_3_N_4_, NbO, NbO/g-C_3_N_4_ and the hybrid material after adsorbing MB (NbO/g-C_3_N_4_-MB) are shown in Figure S1.

**Figure 4:**
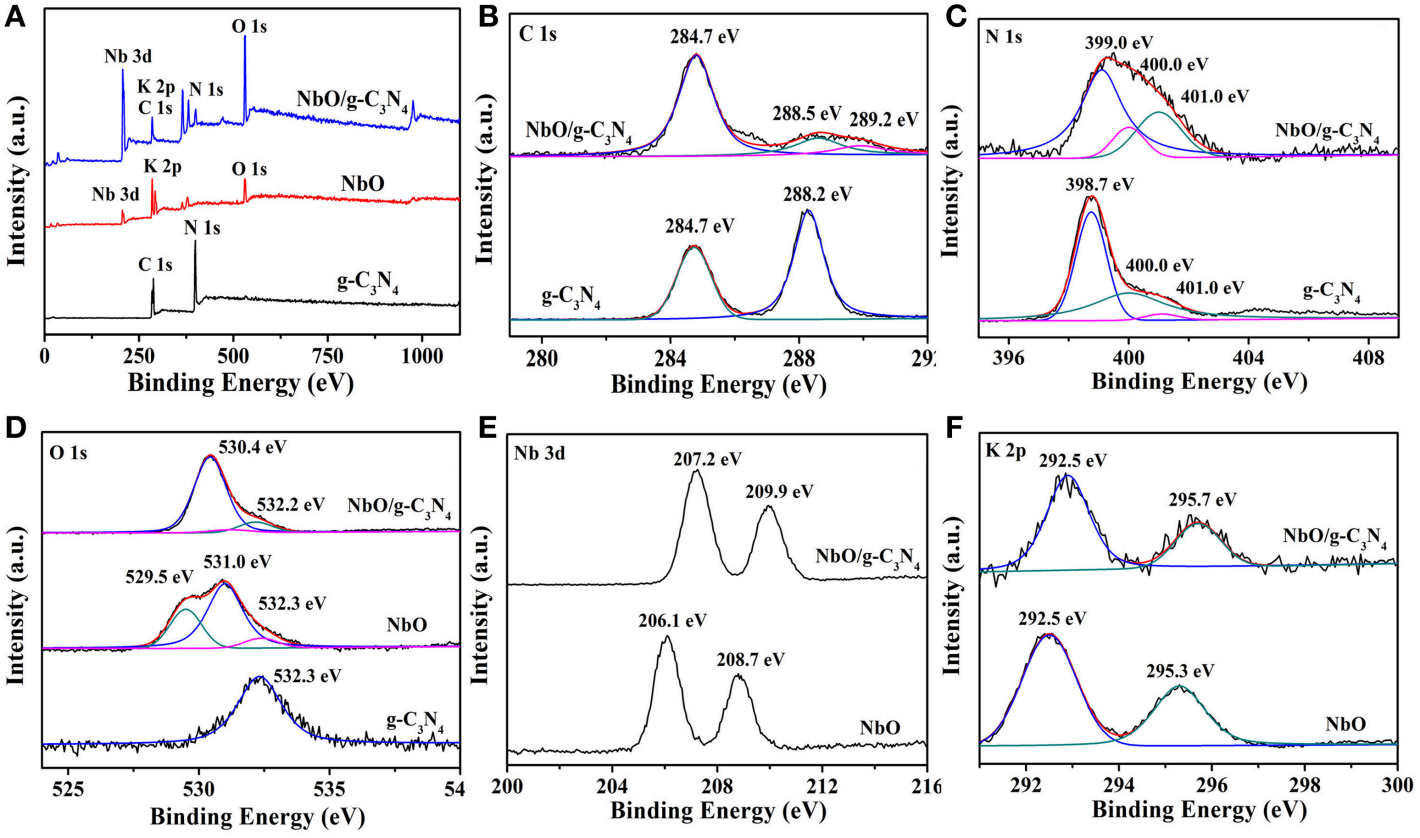
XPS of g-C_3_N_4_, NbO, and NbO/g-C_3_N_4_ in the survey spectra **(A)**, C 1s **(B)**, N 1s **(C)**, O 1s **(D)**, Nb 3d **(E)**, and K 2p **(F)** binding energy regions.

To gain insight into the real composition and chemical states of g-C_3_N_4_, NbO, and NbO/g-C_3_N_4_ hybrid composite, XPS measurements were carried out and the results are exhibited in Figure 4. The full survey spectra of g-C_3_N_4_, NbO, and NbO/g-C_3_N_4_ were displayed in Figure 4A. The presence of Nb, C, N, O, and K elements in NbO/g-C_3_N_4_ indicated that the hybrid composite have all characteristic elements of g-C_3_N_4_ and NbO. It's observed that the main peak centered at 284.7 eV from the C 1s spectrum (Figure 4B) should be ascribed to the sp^2^ C–C or/and C = C bonds, which originates from the adventitious reference carbon on the surface (Zhang et al., 2012). For the g-C_3_N_4_ sample, the C 1s peak centered at 288.2 eV was assigned to the sp^2^ carbon atoms bonded to N (N = C–(N)_2_) in the graphitic structure. As for the C 1s peaks of NbO/g-C_3_N_4_ (288.5 and 289.2 eV) emanate from sp^2^ carbon atoms shift to higher banding energy orientation after through hydrothermal treatment, suggesting that the –NH_2_ groups of g-C_3_N_4_ has been replaced by the –OH groups to form an N = C(N)–OH species. Meanwhile, the peaks also can be affected by the Linqvist unit of NbO due to it has strong electronic pull. Therefore, the results indicated that either terminal oxygen atom from Nb = O or bridge oxygen from Nb–O–Nb groups within Linqvist unit have interactions with (N = C–(N)_2_) group within g-C_3_N_4_ or N = C(N)–OH species resulting from hydrothermal treatment. Three main peaks of N 1s spectra can be seen from Figure 4C, the main peak centered at 398.7 eV should be corresponding to sp^2^ N atoms involved in tris-triazine rings (C = C–N), and other two main peaks ascribed to tertiary nitrogen (N–(C)_3_) (400.0 eV) and N atoms bonded with H atoms (C–N–H) in the aromatic rings (401.0 eV) (Dong et al., 2017). However, the binding energy of the first one in N 1s of the composite (399.3 eV) was found to be slightly bigger than those of g-C_3_N_4_. The similar situation also can be seen in the C 1s and O 1s. Figure 4D shows that g-C_3_N_4_ sample exhibits O 1s peak at 532.3 eV which can be ascribed to the adsorbed water. Three peaks centered at 532.3, 529.5, and 531.0 eV were detected on NbO sample, which is assigned to Nb–O–Nb, Nb–O–H, and adsorbed water, respectively. After through the process of hydrothermal treatment, the peak of NbO/g-C_3_N_4_ in the O 1s has two main peaks. The main peak at 532.2 eV just like g-C_3_N_4_ and NbO, and the other one is narrow peak which results from the binding energy of different oxygen-containing species. Compared with NbO, the changes of Nb 3d and K 2p binding energy in the NbO/g-C_3_N_4_ composite (Figures 4E,F) can further confirm the Linqvist unit of NbO sample has interacted with g-C_3_N_4_ sample.

### Effect of initial dye concentration

**Figure 5:**
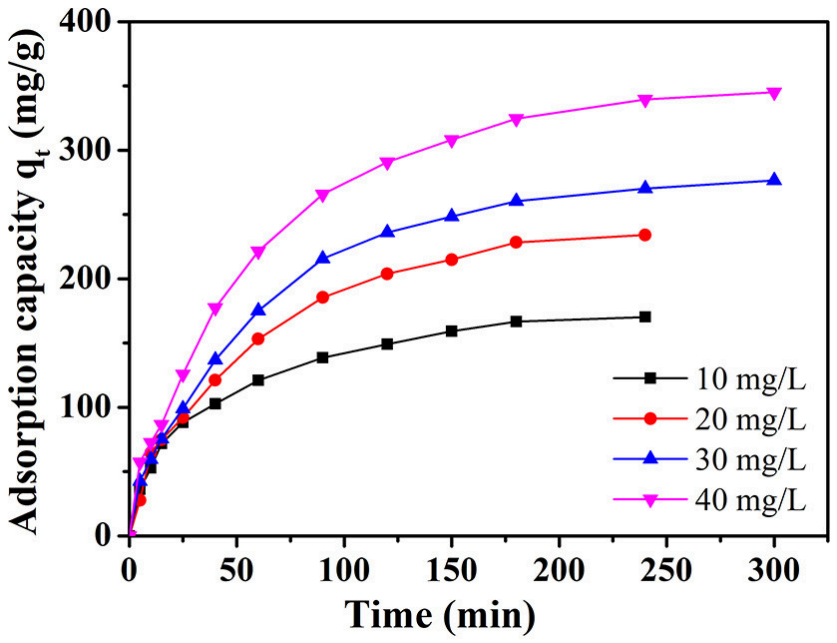
The effect of initial dye concentration on adsorption of MB onto NbO/g-C_3_N_4_. Experimental conditions: T = 30°C, pH = 7.0.

The time-dependent adsorption experiments toward MB with different initial dye concentration (10, 20, 30, and 40 mg L^−1^) were studied on NbO/g-C_3_N_4_ (see Figure 5). The adsorption capacity of the MB increased with time and then reached equilibrium after 240 min. It can be easily observed that the adsorption is quickly increased in the initial stage. And after 90 min, the increasing trend did not stop but slow down gradually. This phenomenon was not hard to explain, which is due to the fact that the most empty surface on the adsorbents were available during the initial stage and the remaining empty surface sites were hard to occupy due to the repulsive forces between the MB molecules on the NbO/g-C_3_N_4_ and the bulk phase (Fu et al., 2015). The equilibrium adsorption capacity of MB with increased initial concentrations shows an increasing trend since the increasing MB concentration can provide an increase driving force to overcome the mass transfer resistance of the dye (Konicki et al., 2013). Hence, it indicates that the initial MB concentration has a great influence on the adsorption process.

### Effect of initial pH

**Figure 6:**
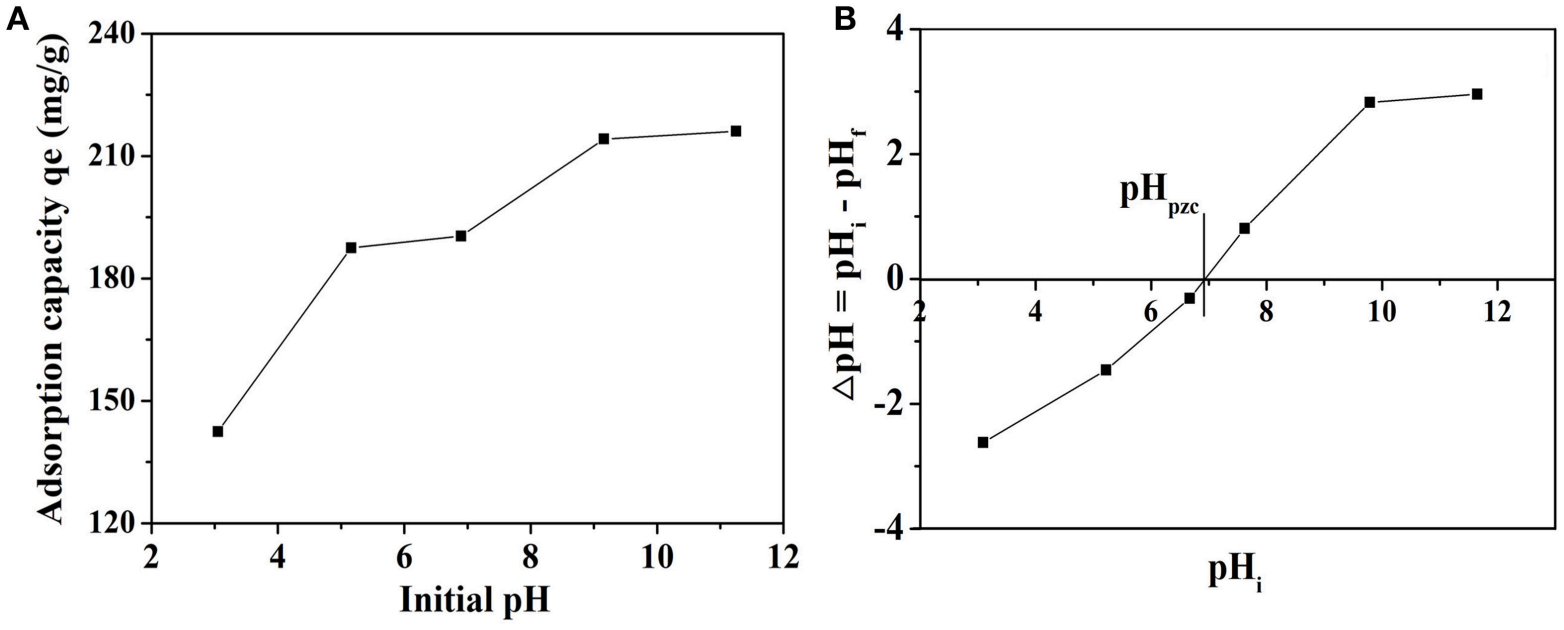
**(A)** The effect of initial pH of dye solution on adsorption of MB onto NbO/g-C_3_N_4_. Experimental conditions: *C*_0, MB_ = 20 mg L^−1^, T = 30°C. **(B)** The pH_pzc_ of NbO/g-C_3_N_4_.

The initial pH of solution usually has a significant effect on MB adsorption process. In order to observe the influence, the adsorption experiments were performed in the pH values ranging from 3.0 to 11.5 at MB concentration of 20 mg L^−1^ and 30°C. The effect of initial pH on the adsorption capacity of MB is given in Figure 6A. The adsorption capacity has a prominent increasing trend from 142.43 to 216.09 mg g^−1^ when the pH of the dye solution was increased over the whole studied range, which is quite similar to the previous research (El Qada et al., 2006; Xu et al., 2017). The increase of adsorbed MB amount with increasing pH might be due to the fact that the concentration of negative charge of the adsorbent increases with increasing pH (Xu et al., 2017). Hence, pH can be seen as an important factor to the adsorption process. The plot of ΔpH vs. pH_i_ was given in Figure 6B and the pH at which ΔpH becomes zero is called pH_pzc_. The pH_pzc_ value for NbO/g-C_3_N_4_ was 6.93 and it is the pH at which the net surface charge on adsorbent is zero. The adsorbent surface has a net positive charge at pH < pH_pzc_, while has a net negative charge at pH > pH_pzc_ (El Qada et al., 2006). It indicates that NbO/g-C_3_N_4_ surface becomes negatively charged when pH > 6.93, and it will be favorite the adsorption of MB in cationic form. Thus, as pH is increased, the adsorbent surface is more negatively charged leading to a biggish attraction between the NbO/g-C_3_N_4_ and MB. Besides, when the pH is under pH_pzc_, the NbO/g-C_3_N_4_ surface is more positively charged, which does not favor the adsorption of dye positive ions because of the electrostatic repulsion.

### Effect of temperature

**Figure 7:**
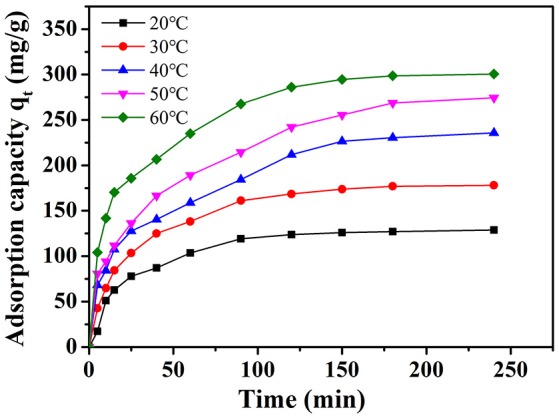
The effect of temperature on the adsorption of MB onto NbO/g-C_3_N_4_. Experimental conditions: *C*_0, MB_ = 20 mg L^−1^, pH = 7.0.

The amount of adsorption of MB on NbO/g-C_3_N_4_ with various temperatures is shown in Figure 7. The adsorption capacity of MB was enhanced with increasing temperature, indicated it is an endothermic process. In order to investigate the thermodynamic parameter, variation of Gibbs free energy (Δ*G*°), enthalpy change (Δ*H*°), and entropy change (Δ*S*°) were determined by the following equations:

(3) $$\begin{array}{r} \Delta G^{{}^{\circ}}=-RTlnK_{a} \end{array}$$

(4) $$\begin{array}{r} K_{\text{a}}=q_{\text{e}}/C_{\text{e}} \end{array}$$

(5) $$\begin{array}{r} \ln\;K_{\text{a}}=\Delta S^{{}^{\circ}}/R-\Delta H^{{}^{\circ}}/RT \end{array}$$

Where *R* and *T* represent the gas constant (8.314 J mol^−1^ K^−1^) and the temperature (K), *K*_a_ is the adsorption equilibrium constant. The enthalpy (Δ*H*°) and entropy (Δ*S*°) can be obtained from the slope and intercept from the Van't Hoff plot of ln *K*_a_ vs. 1/*T* (Figure S2). The values of Δ*G*°, Δ*H*°, and Δ*S*° were calculated and the results are summarized in Table 1.

**Table 1:** Thermodynamic parameters for adsorption MB onto NbO/g-C_3_N_4_.

**Dye concentration (mg L^−1^)**	**Δ*H*° (kJ mol^−1^)**	**Δ*S*° (J mol^−1^ K^−1^)**	**Δ*G*° at temperature (°C) (kJ mol^−1^)**				
			**20**	**30**	**40**	**50**	**60**
20	38.36	150.24	−5.55	−7.14	−8.94	−10.23	−11.48

The negative value of Δ*G*° (−5.55, −7.14, −8.94, −10.23, and −11.48 kJ mol^−1^ at 293, 303, 313, 323, and 333 K, respectively) suggest that the adsorption of MB was spontaneous. The positive value of Δ*H*° (38.3 kJ mol^−1^) confirms that the adsorption of MB on NbO/g-C_3_N_4_ is an endothermic process and the value smaller than 40 kJ mol^−1^ indicates the adsorption is a physical adsorption process (Yao et al., 2010). Furthermore, the positive value of Δ*S*° (150.2 J mol^−1^ K^−1^) indicates that the freedom of the adsorption process was increased in the solid-solution interface. This similar case has also been observed by Fu et al. (2015) and Zheng et al. (2017). Molecules may be restricted on the surface after adsorption and unable to optionally move in three dimensions but as the temperature increased, the motion of molecules obtained free and their disorderliness increased resulting in the entropy increased. Therefore, all the above thermodynamic parameters represent that NbO/g-C_3_N_4_ can be used as an effective adsorbent for MB removal in aqueous solution.

### Adsorption isotherms

The adsorption isotherms can be described as: at a fixed temperature, the adsorbates interact with adsorbent when the adsorption processes of solute molecules reach its equilibrium. Hence, adsorption isotherms are necessary parameters to study the adsorption system. Usually, the Langmuir and Freundlich isotherms were used to depict the equilibrium adsorption. The Langmuir isotherm is based on the assumption that adsorption occurs on a homogeneous surface. Meanwhile, the Freundlich isotherm describes the adsorption process which takes place on a heterogeneous surface. The two models are represented by the following equations:

(6) $$\begin{array}{r} C_{\text{e}}/q_{\text{e}}=1/Q_{0}b+C_{\text{e}}/Q_{0} \end{array}$$

(7) $$\begin{array}{r} \ln\;q_{\text{e}}=\ln\;K_{F}+1/n\ln\;C_{\text{e}} \end{array}$$

Where *C*_e_ represents the equilibrium concentration of MB (mg L^−1^); *q*_e_ and *Q*_0_ represent the adsorption capacity (mg g^−1^) and the maximum adsorption capacity of MB at equilibrium, respectively. The *b* is a Langmuir constant (L mg^−1^) which is related to the adsorption rate. *K*_*F*_ and *n* are Freundlich constants which are related to the adsorption capacity and intensity, respectively. The essential factor of Langmuir isotherm can be expressed in terms of a dimensionless equilibrium parameter (*R*_*L*_), which is calculated by the following equation (Hameed et al., 2007):

(8) $$\begin{array}{r} R_{L}=1/\left(1+{bC}_{0}\right) \end{array}$$

where *C*_0_ (mg L^−1^) represents the highest initial dye concentration and *b* (L mg^−1^) is Langmuir constant. The value of *R*_*L*_ indicates that the type of the isotherm is either favorable (0 < *R*_*L*_ < 1), unfavorable (*R*_*L*_ > 1), linear (*R*_*L*_ = 1), or irreversible (*R*_*L*_ = 0). The calculated value of *R*_*L*_ (0.0056) confirmed that the adsorption of MB on NbO/g-C_3_N_4_ is favorable.

**Figure 8:**
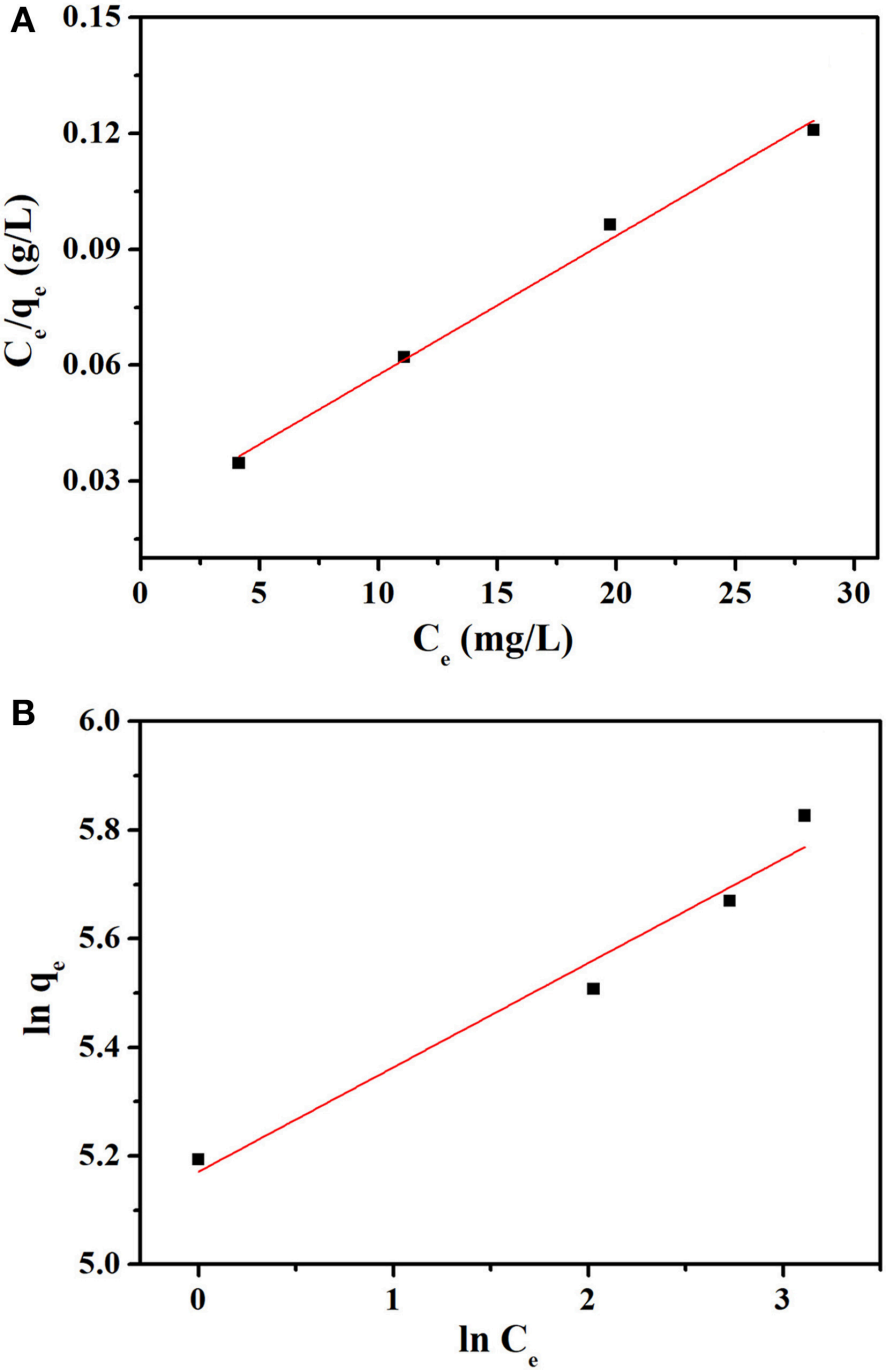
**(A)** Langmuir and **(B)** Freundlich adsorption isotherm of MB onto NbO/g-C_3_N_4_ at 30°C.

The value of *Q*_0_, *b, K*_*F*_, and *n* were determined from the slope and intercept of the linear plot *C*_e_/*q*_e_ vs. *C*_e_ and ln *q*_e_ vs. ln *C*_e_ (Figures 8A,B), and the results are given in the Table 2. The correlation coefficients *R*^2^ (0.9917) of Langmuir model is more close to 1 than that of Freundlich model (0.9501), suggesting the adsorption data fitted well with Langmuir model. In addition, the value of *n* (4.751 L mg^−1^) in the range from 1.0 to 10.0 represented a good adsorption process (Chairat et al., 2008).

**Table 2:** Langmuir and Freundlich parameters for MB on NbO/g-C_3_N_4_.

**LANGMUIR ISOTHERM**	
*Q*_0_ (mg g^−1^)	373.13
*b* (L mg^−1^)	0.4751
*R_*L*_*	0.0056
*R*^2^	0.9917
**FREUNDLICH ISOTHERM**	
*K_*F*_* [(mg g^−1^)(L mg^−1^) ^1/n^]	176.07
*n*	5.2037
*R*^2^	0.9501

Maximum adsorption capacities of various adsorbents applied for adsorbed MB reported in the literatures are displayed in Table 3. Adsorption capacity of NbO/g-C_3_N_4_ in this work is higher than that obtained in most of other studies. This data can be confirmed that NbO/g-C_3_N_4_ is a highly efficient adsorbent in the removal of MB from aqueous solution.

**Table 3:** Comparison of the maximum adsorption capacity of MB onto various adsorbents.

**Adsorbent**	***Q*_0_ (mg g^−1^)**	**References**
Mesoporous carbon nitride	360.8	Zhang et al., 2014
MOFs-235	187	Haque et al., 2011
Graphene	204	Liu et al., 2012
Hydrolyzed carbon nitride	402	Wang et al., 2013
Commercial activated carbon	200	Doke and Yadav, 2014
Fe_3_O_4_/NPC	292.4	Jiao et al., 2016
Halloysite nanotubes	84.23	Zhao and Liu, 2008
Mesoporous hybrid xerogel	144	Wu et al., 2005
Molybdenum disulfide	297	Massey et al., 2016
Hazelnut husk-activated carbon	476.2	Karacetin et al., 2014
NbO/g-C_3_N_4_	373.1	This work

### Adsorption kinetics

**Figure 9:**
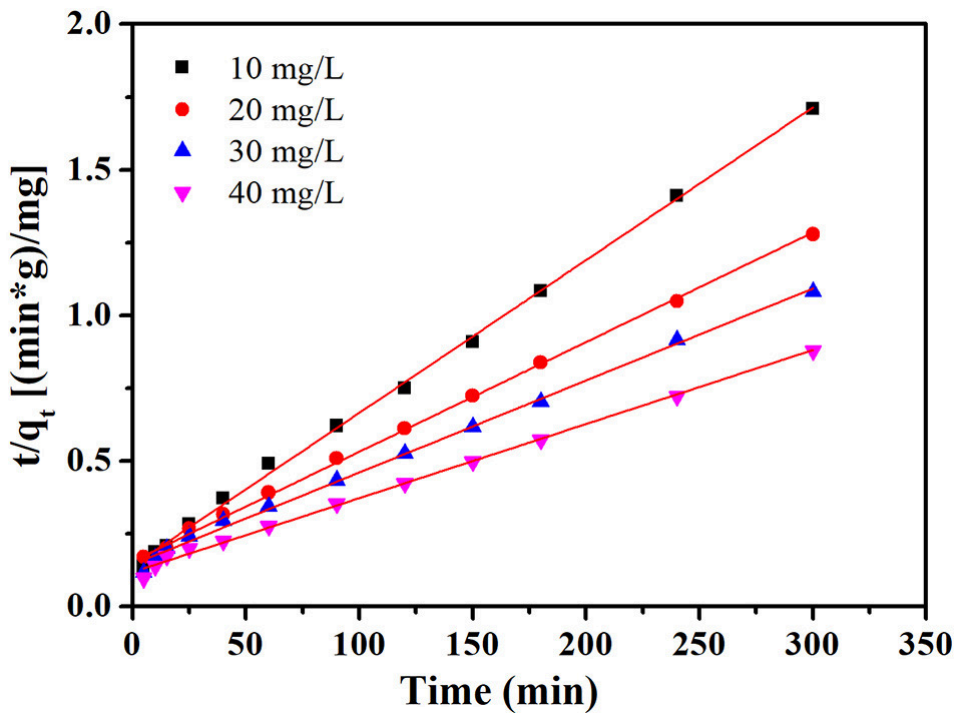
Pseudo second order kinetic model for adsorption of MB onto NbO/g-C_3_N_4_.

In order to completely explore the mechanism of the adsorption of MB on NbO/g-C_3_N_4_, three different kinetic models (pseudo first order, pseudo second order, and intraparticle diffusion model) were used to analyze the kinetic adsorption data. Pseudo first order model is represented by the following equation:

(9) $$\begin{array}{r} \ln\;\left(q_{\text{e}}-q_{t}\right)=\ln\;q_{\text{e}}-k_{1}t \end{array}$$

where *q*_e_ (mg g^−1^) and *q*_t_ (mg g^−1^) represent the adsorption capacity of MB at equilibrium and at time *t*. The *k*_1_ (min^−1^) is the first order rate constant, and the values of *k*_1_ and *q*_e_ were calculated from the slope and intercept of the linear plot ln (*q*_e_ – *q*_t_) vs. *t*, respectively. Meanwhile, the linear plot ln (*q*_e_ – *q*_t_) vs. *t* at the condition of 30°C and initial pH value (7.0) is given in Figure S3. Moreover, pseudo second order model is given by the following equation:

(10) $$\begin{array}{r} t/q_{\text{t}}=1/k_{2}q_{\text{e}}^{2}+t/q_{\text{e}} \end{array}$$

where *k*_2_ (mg g^−1^ min^−1^) is the pseudo second order rate constant. The values of *k*_2_ and *q*_e_ were obtained from the slope and intercept of the plot of *t*/*q*_t_ vs. *t*. The plot of *t* /*q*_t_ vs. *t* at the condition of 30°C and initial pH value (7.0) is given in Figure 9. Furthermore, the intraparticle diffusion model suggested by Weber and Borris was thoroughly studied and the equation can be expressed as follows:

(11) $$\begin{array}{r} q_{\text{t}}=k_{\text{i}}t^{0.5}+C \end{array}$$

where the *k*_i_ (mg g^−1^ min^−0.5^) is the rate constants for intraparticle diffusion model and *C* (mg g^−1^) is the intercept. The value of *k*_i_ was determined from the slope of the plot of *q*_*t*_ vs. *t*^0.5^ (Figure S4).

The relevant parameters (*k*_1_, *k*_2_, and *q*_e_) of MB adsorption on NbO/g-C_3_N_4_ from pseudo first order and pseudo second order models are displayed in Table S1. It can be seen from the plots of the pseudo second order show a good agreement between the experimental and calculated *q*_e_ values for different initial MB concentrations. Moreover, compared to the correlation coefficients of both models, it's obvious that coefficients of pseudo second order model is greater and more near to unity. Hence, it can be concluded that MB adsorption on NbO/g-C_3_N_4_ is described by pseudo second order model.

The experiment data was also analyzed by the intraparticle diffusion model. The graphs of *q*_t_ vs. *t*^0.5^ can be divided into two regions. First region was explained to diffusion of MB molecules from solution to the external surface of NbO/g-C_3_N_4_ and second region was diffusion into pores of NbO/g-C_3_N_4._ As shown in Figure S3, the *k*_i1_ values were bigger than *k*_i2_ values indicating the second region of intraparticle diffusion is a gradually process, which fit in with the parameters of intraparticle diffusion displayed in Table S2. Furthermore, because of the plot did not pass through origin, it was indicated that intraparticle diffusion was not rate-controlling step (Duran et al., 2011). So, the adsorption involved by both intraparticle diffusion as well as diffusion to external surface step (Singh and Pant, 2004; Cheung et al., 2007).

## Conclusions

In summary, a NbO/g-C_3_N_4_ hybrid composite with porous structure was successfully obtained using one-step hydrothermal method. The effects of a variety of operating conditions (initial dye concentration, pH, and temperature) were studied. Results indicated that the adsorption of MB is favored at high pH (216.9 mg g^−1^ at pH = 11.25), and the adsorption capacity of MB increases with increasing initial concentration and temperature. The adsorption data were described by the Langmuir and Freundlich models, and the result suggested the adsorption of MB onto NbO/g-C_3_N_4_ was fitted well with the Langmuir model and maximum adsorption capacity of NbO/g-C_3_N_4_ was found to be 373.13 mg g^−1^. In the adsorption kinetic studies, it was demonstrated that the experiment data was well described by pseudo second order model. Moreover, the intraparticle diffusion model represented that two steps happened during the adsorption process and intraparticle diffusion was not rate-limiting step. The thermodynamic analyses indicated that adsorption MB onto NbO/g-C_3_N_4_ was a spontaneous endothermic process. Hence, it can be expected that NbO/g-C_3_N_4_ can be a new class of adsorbent for removing methylene blue from polluted water effectively.

## Author contributions

QG: carried out all experiments, did data collection, data analysis, and prepared the manuscript; WS: helped analyze the XPS data; YX: helped explain some of the experimental results and revise manuscript; YH: contributed to the scientific interpretation of results and revised the manuscript.

### Conflict of interest statement

The authors declare that the research was conducted in the absence of any commercial or financial relationships that could be construed as a potential conflict of interest.
