# Supplementary material for: A Polyoxoniobate/g-C3N4 Nanoporous Material with High Adsorption Capacity of Methylene Blue from Aqueous Solution
Source: Front Chem. 2018 Jan 31;6:7. doi: 10.3389/fchem.2018.00007 (PMC5797750; doi:10.3389/fchem.2018.00007)
Supplement: Supplementary file 1 [file Presentation1.pdf]

Supplementary Materials

A Polyoxoniobate/g-C_3N_4 Nanoporous Material with High Adsorption Capacity of Methylene Blue from Aqueous Solution

Qiuyan Gan[1], Weilong Shi [2], Yanjun Xing[1*], Yu Hou[1*]

[1]College of Chemistry, Chemical Engineering and Biotechnology, Donghua University, Shanghai, China.
[2]Institute of Functional Nano & Soft Materials, Soochow University, Suzhou, Jiangsu, China.
Correspondence:
houyu@dhu.edu.cn；yjxing@dhu.edu.cn

Supplementary Figures and Tables

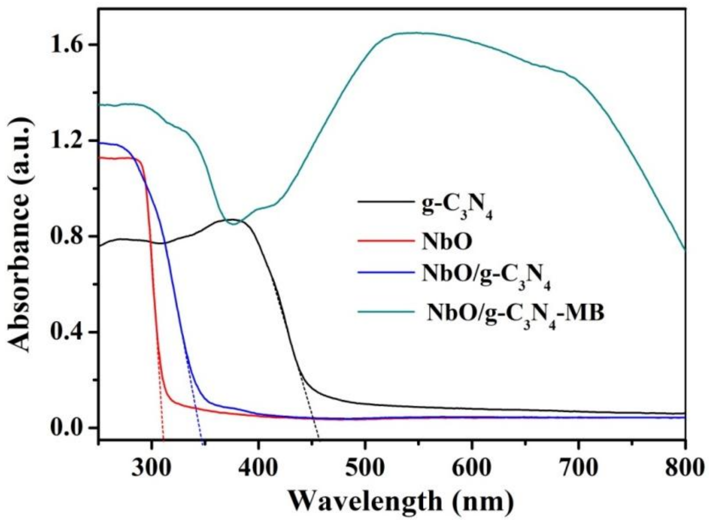

Figure. S1 UV-vis DRS of g-C_3N_4, NbO, NbO/g-C_3N_4 and NbO/g-C_3N_4–MB samples.

UV−vis absorption of g-C_3N_4, NbO, NbO/g-C_3N_4 and the hybrid material after adsorbing MB (NbO/g-C_3N_4–MB) were also measured (Figure. S4). The g-C_3N_4 exhibits absorption in the visible light absorption edge at 457.6 nm, which is corresponding to the band gap of 2.67 eV. After loading NbO onto g-C_3N_4, a significant blue-shift was observed. This change could be due to the interaction of NbO with g-C_3N_4 since NbO has an absorbance band around 311 nm. Moreover, it can be observed that the absorbance band of NbO/g-C_3N_4–MB was different with others. Compared to the absorption edge of MB, the NbO/g-C_3N_4–MB also was blue-shift, indicating that the electrostatic interaction between the hybrid materials and MB.

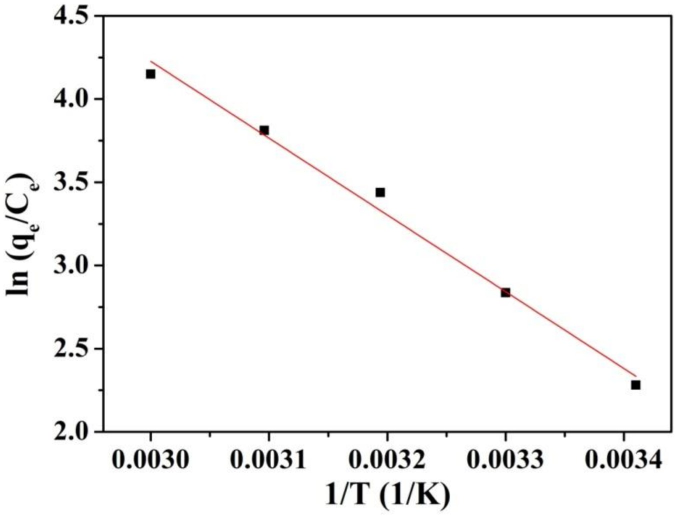

Figure. S2 Van’t Hoff plot for adsorption of MB onto NbO/ g-C_3N_4.

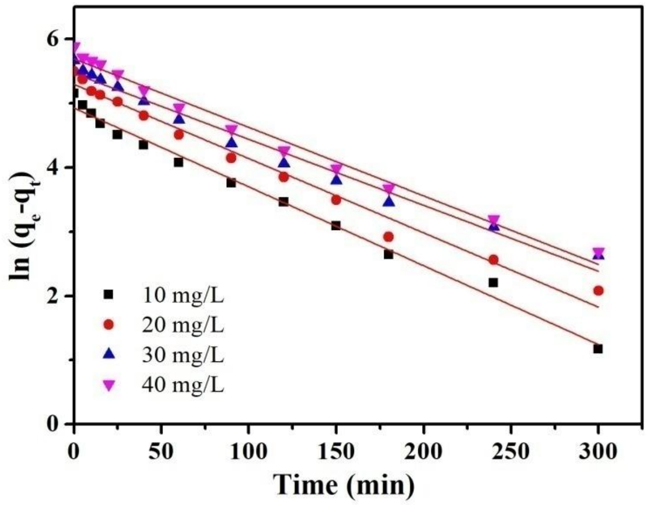

Figure. S3 Pseudo first order kinetics model for adsorption of MB onto NbO/ g-C_3N_4.

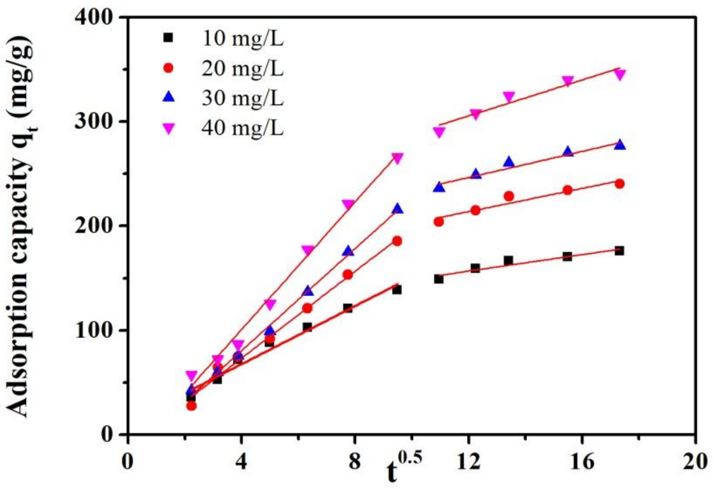

Figure. S4 Intraparticle diffusion model for adsorption of MB onto NbO/ g-C_3N_4.

Table S1 parameters of pseudo first order and pseudo second order models for MB adsorption by NbO/ g-C_3N_4

C_0 (mg L^{-1})	$q_{e\ exp}$ (mg g^{-1})	Pseudo-first-order kinetic model			Pseudo-second-order kinetic model		
		k_1 (min^{-1})	$q_{e\ cal}$ (mg g^{-1})	R^2	k_2 (min^{-1})	$q_{e\ cal}$ (mg g^{-1})	R^2
10	180.3	0.0122	138.4	0.989	0.19×10^{-3}	190.1	0.998
20	244.6	0.0115	200.2	0.983	0.09×10^{-3}	263.8	0.998
30	290.2	0.0102	237.1	0.978	0.07×10^{-3}	312.5	0.996
40	360.0	0.0106	297.7	0.985	0.05×10^{-3}	387.5	0.998

Table S2 Parameters of intrapaiticle diffusion model for MB adsorption by NbO/g-C_3N_4

C_0 (mg L^{-1})	K_{i1} (mg g^{-1} $min^{-0.5}$)	R^2	K_{i2} (mg g^{-1} $min^{-0.5}$)	R^2	C (mg g^{-1})
10	13.91	0.974	3.94	0.887	109.4
20	20.80	0.987	5.54	0.888	147.2
30	24.45	0.996	6.25	0.931	171.5
40	30.55	0.980	8.61	0.910	202.2
